# Books and Authors

**Published:** 1905-09

**Authors:** 


### BOOKS AND AUTHORS.

---

**A Textbook of the Practice of Medicine.** For Students and Practitioners. By **Hobart Amory Hare,** M.D., B.Sc., Professor of Therapeutics and Materia Medica in the Jefferson Medical College of Philadelphia. Author of A Textbook of Practical Therapeutics; A Textbook of Practical Diagnosis, etc. Octavo, 1120 pages, with 129 engravings and 10 full-page plates in colors and monochrome. Philadelphia and New York: Lea Brothers & Co. 1905. (Cloth, $5.00; leather, $6.00; half morocco, $6.50, net prices.)

A new textbook on medicine at this time will receive hearty welcome, and especially will this author be congratulated on all hands by his colleagues and pupils upon his appearance in this field. He is thoroughly well known to the medical profession of the world through his works on diagnosis and therapeutics that have gone through many editions, as well as by many other articles on medical subjects that have appeared during the past twenty years.

The arrangement of the treatise is excellent and its material is presented in accessible form. The section on infectious diseases is the most complete yet presented in a single volume treatise on medicine. We like the subheads to the section on diseases of the nervous system—diseases in which the chief manifestations are in the brain and its membranes, in the spinal cord or its membranes, in the nerves, in the muscles—which serve to subdivide and classify the several groups of nervous disease in a clear and logical manner, and the section properly closes with functional nervous diseases.

In view of the acquirement of islands in the tropics, it is quite important that students of medicine as well as medical men should be made familiar with diseases peculiar to the tropics. In no general treatise on medicine has this subject been so clearly and fully discussed as in this; besides, in none has its importance been made so manifest.

The tuberculosis problem is another topic that Hare handles with a perspicacity born of scientific training, and one with which he exhibits thorough acquaintance. Its importance to the medical student and junior physician is not second to that of any other in the field of medicine, and the latest views concerning methods of invasion and prophylaxis,—the two most important questions related to tuberculosis,—are amply dealt with.

And so we might go on pointing out features of excellence in this treatise, until the reader would be justified in crying out "peccavi," for it abounds in such from cover to cover. The principal points to be considered in contemplating the purchase of a new textbook on medicine are: is it written by a clinician of experience? Is the author an accurate observer who presents his facts with due regard for their precise value? Is he a teacher of first rank, capable of imparting his knowledge to those who seek instruction? Has he given the latest and best digested thought on the moot questions of general practice? Has he presented a work that teachers will be apt to adopt as a textbook for their classes?

These interrogatories all may be affirmatively answered in regard to Hare's treatise. It represents the essentials of medicine as taught today in the best schools and is likely to be generally put upon their lists of textbooks.

---

**Acute Contagious Diseases.** By **William M. Welch,** M.D., Consulting Physician to the Municipal Hospital for Contagious and Infectious Diseases; Diagnostician to the Bureau of Health, etc., Philadelphia, and **Jay F. Schamberg,** A.B., M.D., Professor of Dermatology and of Infectious Eruptive Diseases, Philadelphia Polyclinic; Consulting Physician to the Municipal Hospital for Contagious and Infectious Diseases, and Assistant Diagnostician to the Philadelphia Bureau of Health, etc. Octavo, 781 pages. Illustrated with 109 engravings and 61 full-page plates. Philadelphia and New York: Lea Brothers & Co. 1905. (Cloth, $5.00; leather, $6.00; half morocco, $6.50, net.)

These authors enjoy special opportunities for the study of acute infectious diseases. Dr. Welch is the diagnostician to the bureau of health of the city of Philadelphia, and is consulting physician to the municipal hospital for contagious and infectious diseases. Dr. Schamberg is the assistant diagnostician to the bureau of health, consulting physician to the same hospital, and professor of dermatology at the Philadelphia polyclinic. They are, therefore, experts on the diseases dealt with in this book.

The study of vaccinia is inseparably connected with that of smallpox and is given first place in this book. It is the ablest exposition of the great discovery of Jenner that we have seen; historically, it is most interesting, and scientifically, it is superb. The illustrations showing the various stages and complications of the vaccine disease have never been excelled and the statistics and other data concerning revaccination are reassuring with reference to the prophylactic qualities of vaccinia. The sceptic should read and be convinced.

The importance of differential diagnosis in suspected smallpox cannot be too strongly accentuated. Mistakes in the early stages of the exanthemata have frequently happened and sometimes even experts have been deceived. This treatise, by its text and illustrations will aid greatly in preventing errors of this kind. Some of the plates carry the subject through the several stages of the disease from the first appearance of the eruption to desquamation and final recovery; others exhibit convincing evidence of the immunising effect of successful vaccination; while others, again, give interesting exhibitions of smallpox complicated with other eruptive diseases. It is altogether a most gratifying portrayal of the several phases of the ever to be dreaded smallpox, now happily under control when proper vigilance is exercised.

Scarcely less interesting is the delineation of scarlet fever which, next after diphtheria, is the disease mothers most dread on behalf of their children. Here is an opportunity for the exercise of diagnostic acumen, and so to prevent, oftentimes, the unnecessary spreading of the disease.

Other diseases are dwelt upon with perspicuity—namely, chickenpox, measles, diphtheria and typhoid fever, making the book as complete as may be in text, illustrations and general dress.

---

**Surgical Diagnosis.** A Manual for Practitioners of Medicine and Surgery. By **Otto G. T. Kiliani,** M.D., Surgeon to the German Hospital, New York. Octavo, pp. xviii.-449. Illustrated by 59 full-page plates and engravings. New York: William Wood & Co. 1905. (Price: cloth, $4.50; h. m., $5.50, net.)

The object of this book appears to be to instruct the attending physician as to the proper time to invoke the aid of a surgeon. Ordinarily, this cannot be a difficult problem in injuries of the extremities or cavities of the graver character. In secondary conditions or in so-called surgical diseases, however, it becomes a serious question to decide, and the decision must be rendered with promptitude. Moreover, it becomes necessary to send for the right man. A general surgeon, for example, is not required to operate on the eye, ear, nose, or throat, nor would a genito-urinary surgeon be needed to make a Cesarean section. Who would think of sending for the surgeon skilled in fractures, dislocations, and amputations, to make an abdominal section for some intricate condition within the pelvic or abdominal cavities? Hence, the general practitioner, the man who sees the patient first or who has been in charge from the beginning, must needs diagnosticate not only the disease or injury but also the surgeon, for the personal equation is to be reckoned with very considerably in the conditions under consideration.

Kiliani, in the preparation of this treatise, has for his object not only a mere description of symptoms, but also a desire to make clear their meaning and value. How well he has succeeded depends upon the intelligence of his audience. One will profit, and another will fail to improve by his methods. The author was a pupil of Volkmann and Schede, and has had experience as a clinician and as an operator. His service at the German hospital, New York, affords ample opportunity to gather material on which to base such a treatise. So much for the author.

As to the book itself, we think most of its claims are justified. It may be regarded as a revised and enlarged edition of Ranney's treatise on surgical diagnosis, published twenty-five years ago and now out of print. The arrangement of the book in anatomical sequence, beginning with the head and running downward through the body, is to be commended, as also is the introduction of tables to facilitate differential diagnosis.

The text, for the greater part, is broken up into small paragraphs, and the sentences are quite often epigrammatic in style. Lavish use of heavy-faced type is made in the pages, which serves to accentuate the author's meaning, but which, on the other hand, sometimes mars the appearance of a page by overdoing the method. On the whole, however, the book is a handsome one, —its letter-press, quality of paper, size of page and general make-up all contributing to render it a striking volume. It will easily take a permanent place on the shelves of present-day medical men.

---

**A Textbook of Medical Chemistry and Toxicology.** By **James W. Holland,** M.D., Professor of Medical Chemistry and Toxicology, and Dean, Jefferson Medical College, Philadelphia. Octavo volume of 600 pages, fully illustrated, including 8 plates in colors. Philadelphia and London: W. B. Saunders & Co. 1905. (Cloth, $3.00 net.)

Professor Holland has been known to the medical world for some years as one of the foremost teachers of physiologic chemistry, and we deem it opportune that he has prepared a book embodying his teachings. It is important that the student should be taught properly in the beginning, else much of his later instruction will be vain. This author is not only familiar with his subject, but he knows how to impart his knowledge to the pupil, whether he be the novitiate or the more advanced learner.

The definitions herein given are simple, concise, and easily acquired. The author has introduced topics which the student of physiologic chemistry must acquire as preliminary to the subject proper, though not embodied in most medical textbooks, such as the equilibrium of equations, mass-action, cryoscopy, osmotic pressure, dissociation of salts into ions, the effects of ionisation upon electric conductivity, and the relationship between purin bodies, uric acid, and urea.

Toxicology is so intimately associated with chemistry, that much space is necessarily devoted to the consideration of that topic. A knowledge of the doctrine of poisons is essential to every physician, and this he must acquire during his student life proper, and from an instructor who knows how to teach this subject, else he will make a sad showing in after years when it may become necessary to make clinical use of his talents.

It is quite unnecessary at the present time to present a critical or technical review of any work on chemistry; it is quite likely that any such presentation of this or another treatise would fall as a dead weight on the eyes of medical journal readers. Nevertheless, we may speak of one or two sections of Holland's book, because of the importance of the topics. The first is the chemistry of digestion; the second is the blood; the third is the urine; and, finally the chemistry of milk. These are all presented from the clinical viewpoint and are seldom as clearly set forth in so few words, comparatively speaking, as in this book.

A good index has been supplied to this excellent treatise, which contributes very materially to its usefulness. Indeed, we are of the opinion that no more practical treatise on medical chemistry and toxicology has been offered to the student or practitioner of medicine.

---

**Chemical and Microscopical Diagnosis.** By **Francis Carter Wood,** M.D., Adjunct Professor of Clinical Pathology, Columbia University. New York. Octavo, pp. xxiv.-745. With 188 illustrations and 9 colored plates. New York and London: D. Appleton & Co. 1905.

So much importance attaches to chemistry and microscopy as diagnostic aids that it is essential for the clinician of the present to be familiar with their application to the study of the blood, the secretions, and the excretions of the body.

The author of this work is a pathologist of distinction and presents his studies from the laboratory standpoint. His technic is described in plain terms, readily understandable by the person without technical training, but who has some familiarity with chemistry and the use of the microscope. The blood researches of late have cleared up many doubtful conditions, and sometimes the careful examination of that fluid can alone determine the true disease. Wood has given the best that is yet known on this subject and has dealt with it from the viewpoint of the clinician.

The sputum is another topic of importance, and its study often enables the clinician to make early diagnosis in pulmonary tuberculosis. The author is clear in his teachings on this subject and his methods again are entitled to praise for their simplicity and completeness. Woods's studies of the urine are about the best yet delineated, and what he says on the subject is entitled to great consideration. Here again, the clinician will find aid when groping in the dark, much of the material being quite new.

The chemistry of the gastric contents and of the feces is set forth to its fullest extent; indeed, there is scarcely a fluid, an exudate, a waste product or a solid that is not dealt with in this book in the light of the latest knowledge, as far as their studies can be made useful in the diagnosis of disease. The index, comprising forty pages, is most complete, which is an item of great significance. We regard this as one of the most important medical books that has appeared of late, and venture the opinion that it will not be long before it will be found in the hands of every laboratory worker, whether teacher or pupil.

---

**The Urine and Feces.** A Practical Manual on the Urine and Feces in Diagnosis. By **Otto Hensel,** Ph.G., M.D., Bacteriologist to the German Hospital, New York, and Richard Weil, A.M., M.D., Pathologist to the German Hospital, New York, in collaboration with Smith Ely Jelliffe, M.D., Ph.D., Instructor in Pharmacology and Therapeutics, Columbia University, New York. Octavo, 334 pages, illustrated with 116 engravings and 10 colored plates. New York and Philadelphia: Lea Brothers & Co. 1905. (Cloth, $2.75 net.)

The output of works dealing with urinary analysis seems to have reached a climax in this book which is designed with a fixed purpose in view,—the production of a compact, handy and trustworthy guide to the study of the urine and the feces. The aim has been successfully accomplished and a book has been given the profession which leaves nothing to be desired and gives much to be thankful for. Combining all the good points of larger and bulkier works on analysis of urine and urinary diagnosis, it adds the feces—a heretofore much neglected field. The very excellent arrangement of the divisions of analytic work is welcome. There is an apparent and refreshing directness in the presentation of the normal and abnormal constituents and in the tests and findings. What strikes one as particularly useful is the page and a half devoted to urinary diagnosis. In this space is given all the information in a more comprehensive form, that other writers have struggled with in an entire chapter—and with less satisfaction.

The portion of the book devoted to the feces is equally satisfactory. Nothing is overlooked—no point which could possibly be of aid to the general practitioner is ignored. Taken from cover to cover there can be no hesitancy in according it unstinted praise,—and declaring it one of the best, most complete and most satisfactory book which could reach the table of the every day practitioner. He will find much good in even a cursory reading of it. N. W. W.

---

**Gynecology.** Medical and Surgical Outlines for Students and Practitioners. By **Henry J. Garrigues,** A.M., M.D., Gynecologist to Saint Mark's Hospital, New York. Octavo, pp. xxlll.-461. With 343 illustrations. Philadelphia and London: J. B. Lippincott Co. 1905. (Price, $3.00.)

It is well that a practical work on gynecology by this author should be presented for the guidance of students and practitioners. Garrigues has been known to the profession of medicine throughout the world as an author for many years. His two books,—one on diseases of women and the other on obstetrics,—have been quite generally adopted as textbooks in the colleges, and each has also found favor with a very large number of practising physicians.

This book is particularly designed for students yet in college as well as for that large class of general or family physicians who must needs practise the minor details of gynecology. It aims to outline the entire system of gynecology, laying special stress on minor operations and details of technic which practising physicians should understand. Garrigues's description of the examination of a patient, including all the various steps leading up to the exploration of the pelvis and its several organs and structures, is concise and yet sufficiently ample for such a treatise.

The author's suggestions as to treatment in general, including hemostasis, instrumentation, care of instruments, and many other topics that must always be familiarised in the beginning, are well arranged and systematically applied. He has given some excellent instruction in regard to the diseases of the perineum, under which term he is pleased to include injuries as well as all pathologic conditions of that region. Under the general heading, diseases of the uterus, Garrigues also includes everything pathologic relating to that organ including every operative procedure from curetment to hysterectomy.

The entire volume is made up of useful instruction by an experienced teacher, upon the essential points belonging to gynecology. It is compact, well illustrated and makes up into one of the best intermediate books we are familiar with,—best for the student, best for the general practitioner.

---

**Lea's Series of Medical Epitomes.** Edited by **Victor C. Pedersen,** M.D. A Manual of Medical Diagnosis. By Austin W. Hollis, M.D., Attending Physician to Saint Luke's Hospital, New York. Duodecimo, 319 pages, with 13 illustrations. New York: Lea Brothers & Co. 1905. (Cloth, $1.00 net.)

The publishers in their circular notice of this number have epitomised the subject without exaggeration, and even better than we can state it, in the following language:

> For the student preparing for examinations, the candidate who is brushing up for appearance before his state board, or for the physician who wishes a handy volume to slip into his pocket or under the cushion of his carriage seat, so that at odd moments he may refresh his memory on forgotten details or post himself on the most recent knowledge on any medical subject, the volumes of this series have proved to be far in advance of any previous attempt. Dr. Hollis's volume on medical diagnosis, just published, is the fifteenth of the series. It naturally does not claim originality, but it embodies an earnest effort to give a clear, accurate, compendious covering of the essentials of its subject, presented with a due sense of the relative importance of its various branches.

We are pleased to express our entire approbation of the foregoing and to add that we appreciate the growing favor of these time-savers and useful aids to the memory.

**A System of Physiologic Therapeutics.** A Practical Exposition of the Methods, other than Drug-giving, useful in the Treatment of the Sick and in the Prevention of Disease. Edited by **Solomon Solis Cohen,** A.M., M.D., Professor of Clinical Medicine at Jefferson Medical College, Philadelphia. Vol. XI. Serum-Therapy; Organotherapy; Blood-letting, etc.; Radium; Principles of Therapeutics; X-ray Therapy; Digest and Therapeutic Index to all volumes. By Joseph McFarland, M.D., Philadelphia; O. T. Osborne, M.D., New Haven; Frederick A. Packard, M.D., Philadelphia; Samuel G. Tracy, M.D., New York; The Editor, and R. Max Goepp, M.D., Philadelphia. Octavo, pp. 388. Illustrated. Philadelphia: P. Blakiston's Son & Co. 1905. (Complete set, cloth, $27.50 net.)

The concluding volume of this remarkable work will be welcomed by those who possess or are familiar with the preceding numbers of the series. The distinguished editor has spent twenty years of thought and five years of work on the system of physiologic therapeutics, which is now brought to a close as far as its publication is concerned; but it will live for ages, let us hope, in the minds and on the shelves of those who believe there are many excellent remedies besides those kept in the drug shops.

This volume contains serum therapy, by Joseph McFarland; organotherapy, by Oliver T. Osborne; radium, thorium, and radioactivity, by Samuel G. Tracy; counterirritation, external applications, bloodletting, by Frederick A. Packard; and an outline of the principles of physiologic therapeutics in particular, by the editor.

An addendum on *x*-ray therapy is placed next after Dr. Osborne's article; an index-digest of therapeutic measures embracing the contents of the eleven volumes, and a separate index of the eleventh and last volume bring to a close a work that will remain a monument to the skill, erudition, and industry of Solomon Solis Cohen.

---

**The Johns Hopkins Hospital Reports.** Vol. XII. Baltimore: The Johns Hopkins Press. 1904.

The contents of this report includes,—the connective tissue of the salivary glands and pancreas with its development in the glandula submaxillaris, by Joseph Marshall Flint; a new instrument for determining the minimum and maximum blood pressure in man, by Joseph Erlanger; metabolism during pregnancy, labor, and the puerperium, by J. Morris Slemons; an experimental study of blood pressure, and pulse pressure in man, by Joseph Erlanger and Donald R. Hooker; typhoid meningitis, by Rufus I. Cole; the pathological anatomy of meningitis due to bacillus typhosus, by William G. MacCallum; a comparative study of white and negro pelves, with a consideration of the size of the child and its relation to presentation and character of labor in the two races, by Theodore F. Riggs; and renal tuberculosis, by George Walker.

The blood pressure study is the most elaborate yet published and will attract the attention of every clinician. The study of white and negro pelves contains much of interest to obstetricians. Indeed, it maybe said of the whole group of papers that they embrace subjects of practical value to every teacher.

The book contains 548 quarto pages printed on tinted book paper, in paper cover, being uniform with the preceding reports from this institution.

---

**The Practical Medicine Series of Year Books.** Ten volumes. Issued under the general editorial charge of **Gustavus P. Head,** M.D., Professor of Laryngology and Rhinology in the Chicago Post-Graduate Medical School. Series 1905. Volume I. General Medicine. Edited by Frank Billings and J. H. Salisbury. Volume II., General Surgery. Edited by John B. Murphy. Chicago: The Year Book Publishers. (Price, $1.00 and $1.50; entire series, $5.50, payable in advance.)

I. The medical gleanings of the year as collated in the first volume are most interesting, and will serve an excellent purpose by placing in the hands of the general practitioners the latest and best thought on some of the topics of greatest importance in everyday practice. Dr. DeLancey Rochester, of Buffalo, is liberally quoted on the early diagnosis and symptoms of artero-sclerosis, a subject upon which he speaks with authority.

II. The surgical volume is full of interest and contains a fund of well-selected material. Especially is the section on intestinal surgery both attractive and instructive. Walter B. Dorsetts's preparation of the patient for abdominal section is given with considerable detail; also J. A. MacLeod's treatment before operation (BUFFALO MED. JOUR., August, 1904,) is set forth with precision. These two volumes start the series of 1905 in a most satisfactory manner and will prove useful to a busy profession.

---

**Malformations of the Genital Organs of Women.** By **Ch. Debierre,** Professor of Anatomy in the Medical Faculty at Lille. Duodecimo, pp. 182. With 85 illustrations. Translated by J. Henry C. Simes, M.D., Philadelphia: P. Blakiston's Son & Co. 1905. (Price, $1.50.)

Debierre has entered a new field in a most searching manner in presenting this book, which is most interesting from both teratologic and anatomic standpoints. The subject is complete in every respect and no abnormality of the female genital anatomy, however slight, has been overlooked. Each section of the subject is introduced with anatomic, histologic and physiologic descriptions and the anomalies and malformations are then described and illustrated. The pictorial portions are extremely well done. Most interesting is the chapter on the vulva, especially in its relation to double pygopagic monsters—those joined back to back. Classic specimens of this type referred to and minutely described are Judith-Helene and Millie-Christine, the latter monster having been made familiar in this country by exhibition some years ago.

